# A Split‐Scar Study: Study of Surgical Scar Using Exosomes

**DOI:** 10.1111/jocd.70756

**Published:** 2026-02-26

**Authors:** Kyung Hwan Jeong, Kui Young Park, Dong‐Woo Jung

**Affiliations:** ^1^ C.LILY Facial Rejuvenation Center Beijing China; ^2^ Department of Dermatology Chung‐Ang University, College of Medicine, Chung‐Ang University Hospital Seoul Republic of Korea; ^3^ Honesty Plastic Surgery Clinic Seoul Republic of Korea

**Keywords:** autologous costal cartilage revision rhinoplasty, cicatrix, exosomes, postoperative scar management, regenerative medicine, scar improvement, wound healing

## Abstract

**Background:**

Exosomes have emerged as a promising therapeutic agent for various dermatological conditions such as acne, atopic dermatitis, and wound healing. This study aims to evaluate exosome's efficacy in improving postoperative scars.

**Methods:**

Ten patients underwent revision rhinoplasty with autologous costal cartilage, each with a 3 cm anterior chest incision scar were enrolled. Scars (mean 4.3 months postoperatively) were divided into medial and lateral halves; one half was treated with ASC‐Exosome (experimental) and the other with hyaluronic acid (control). Outcomes were assessed using the Vancouver Scar Scale (VSS) and the Patient and Observer Scar Assessment Scale (POSAS).

**Result:**

The experimental side treated with ASC‐Exosome demonstrated improvements compared to the control side. Pigmentation improved significantly in the Observer Scar Assessment Scale (OSAS) group from week 3 onward, though no consistent changes were observed on the VSS. Pliability showed significant improvement in both OSAS and VSS, beginning at week 3 and persisting through week 8. Relief also improved in the OSAS group from weeks 4 to 8. While no vascularity differences were detected, one‐year follow‐up photographs confirmed superior improvements in scar height and thickness on the exosome‐treated side. Patient Scar Assessment Scale (PSAS) indicated significant improvements in scar color by week 4, stiffness by week 3, and thickness by week 2. Irregularity showed significant differences at week 4 and week 8. No significant differences were noted in pain or itching between the sides.

**Conclusions:**

Exosomes significantly improved scar quality, particularly pigmentation, pliability, and relief, and can represent a valuable option for postoperative scar management.

## 1 Introduction

Postoperative scar management has employed a range of treatment strategies aimed at preventing hypertrophic scar formation [1], including silicone gels or sheets [2], steroid injections, botulinum toxin [3], and laser therapy [4]. These approaches primarily target inflammation [5] and reduce fibrosis [6] during the wound healing process.

Because scar maturation is a prolonged process, treatment outcomes can vary depending on the timing of intervention [7]. Recent studies suggest that early interventions, particularly during the early wound healing stage [8], yield more favorable clinical outcomes in scar management [9, 10]. Techniques such as polydeoxyribonucleotide [11] and polynucleotide injections have shown promise in promoting wound healing at this critical early stage, thereby reducing the risk of hypertrophic scar development [12, 13].

More recently, exogenous exosomes from adipose‐derived stem cells (ASC‐exosomes) have demonstrated substantial potential in enhancing angiogenesis [14], promoting skin regeneration [15], inhibiting the production of inflammatory mediators and activation of inflammatory cells [16], and facilitating wound healing [17]. Consequently, they help reduce excessive fibrosis and scar formation [18]. While exosomes have garnered attention for cosmetic dermatology treatment, ASC‐exosomes have been particularly effective in modulating inflammatory mediators [19], especially in aesthetic challenges related to skin resurfacing [20].

However, research on the use of exosomes for surgical scars remains limited. This study aims to investigate the effects of exosomes on the improvement of postoperative scars in patients undergoing revision rhinoplasty using autologous costal cartilage, specifically focusing on a scar approximately 3 cm in length on the anterior chest.

## 2 Materials and Methods

### 2.1 Subjects

This prospective, double‐blind, randomized, split‐face comparative study was conducted from June 2024 to November 2024 to evaluate the clinical efficacy of ASC‐exosomes for postoperative scars, in accordance with the principles outlined in the Declaration of Helsinki. The study included 10 patients, consisting of three men and seven women. The mean age was 30.6 ± 3.3 years. The mean postoperative period at the start of the study was 4.3 months (Table 1).

**TABLE 1:** Demographic and clinical characteristics of the study group.

Patients ID	Sex	Age(yr)	Study start point (Months)
1	M	33	10
2	F	33	3
3	M	32	4
4	F	32	11
5	F	27	6
6	F	33	1
7	M	29	2
8	F	26	2
9	F	34	6
10	F	27	4
Mean	M:3 / F:7	30.6 ± 3.3	4.3

Inclusion criteria were patients who had undergone revision rhinoplasty using costal cartilage, presenting with linear scars on the anterior chest area. Exclusion criteria included patients who developed wound dehiscence during the study period.

### 2.2 Design of Study

**FIGURE 1:**
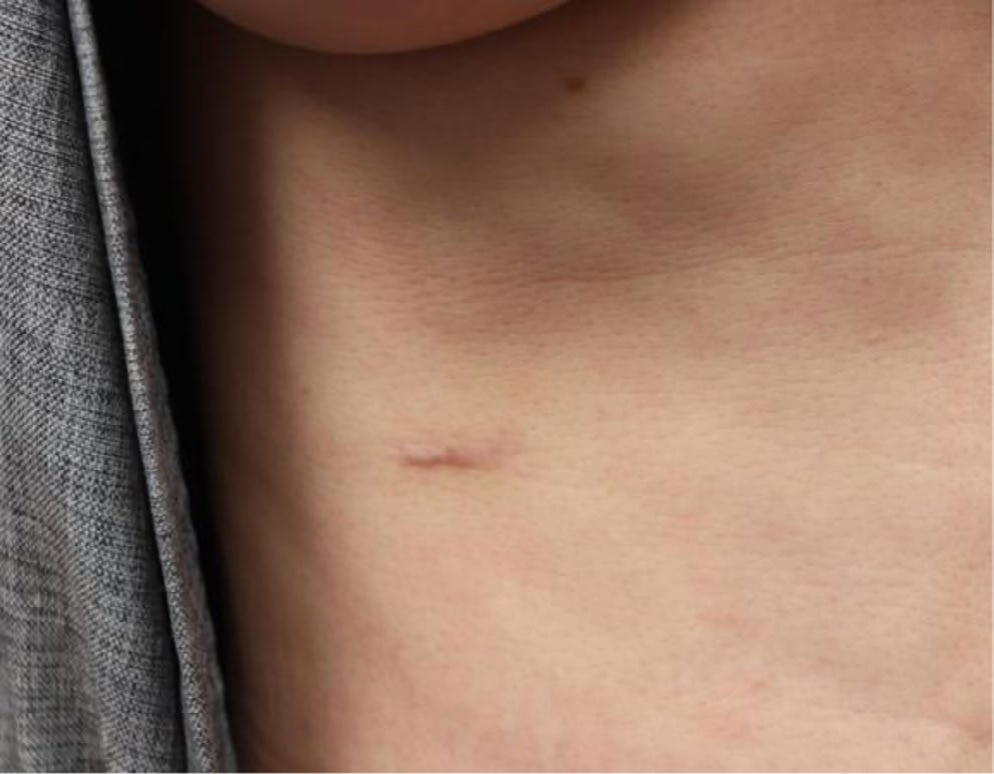
Scar photo after costal cartilage harvest on chest.

Approximately 3 cm incision sites were targeted (Figure 1). The postoperative scar was divided into medial and lateral halves, with the experimental and control sides randomly assigned to each half. The experimental side received an application of 1 cc of ASC‐exosomes formulation (ASCE+ SRLV, ExoCoBio Inc., Seoul, Republic of Korea), while the control side received an application of 1 cc of hyaluronic acid (HA). The ASC‐exosome product contained exosomes obtained from the conditioned medium of human adipose‐derived stem cells (ASCs), processed using ExoSCRT technology as previously described [18, 20].

To facilitate exosome absorption, micro‐needle radio frequency (SCARLET, ViOL, Republic of Korea) was applied in two 2 passes (intensity 7, depth 2.4 mm, delay 0.4 s, duty on 200) before application of exosomes and HA. Both agents were applied topically.

Treatments were administered weekly for eight consecutive weeks, and clinical outcomes were assessed at each visit using standardized photographs and video recordings. To evaluate long‐term outcomes, additional follow‐up photographs were obtained at post treatment one year. Although quantitative evaluation was not performed at this time point, the images allowed visual confirmation of sustained clinical effect.

To ensure double‐blind design, treatment was performed by a blinded physician (Dr. Jung), and evaluation was independently conducted by another blinded physician (Dr. Jeong) using standardized photographs and video recordings.

### 2.3 Measurement

Weekly assessments were conducted using the Vancouver Scar Scale (VSS) and the Patient and Observer Scar Assessment Scale (POSAS) (Tables 2 and 3). Results were averaged and statistically analyzed to ensure objective significance. Data analysis was conducted using IBM SPSS Statistics for Windows, version 22.0 (IBM Corp., Armonk, NY, US). The Mann–Whitney U test and Fisher's exact test were used. In all statistical analyses, a *p*‐value of less than 0.05 was considered statistically significant.

**TABLE 2:** Modified Vancouver scar scale.

Modified Vancouver scar scale		
Pliability	0	Normal
	1	Supple
	2	Yielding
	3	Firm
	4	Adherent
Height	0	Normal
	1	1 ~ 2 mm
	2	3 ~ 4 mm
	3	5 ~ 6 mm
	4	> 6 mm
Vascularity	0	Normal
	1	Pink
	2	Red
	3	Purple
Pigmentation	0	Normal
	1	Slightly
	2	Moderately
	3	Severely

**TABLE 3:** The Patient and observer scar assessment scale.

Observer scar assessment scale												
	Normal skin	1	2	3	4	5	6	7	8	9	10	Worst scar
Vascularization												
Pigmentation												
Thickness												
Relief												
Pliability												
Patient scar assessment scale												
	No	1	2	3	4	5	6	7	8	9	10	Yes
Painful												
Itching												
	Normal skin	1	2	3	4	5	6	7	8	9	10	Very different
Color												
Stiff												
Thickness												
Irregular												

## 3 Results

**FIGURE 2:**
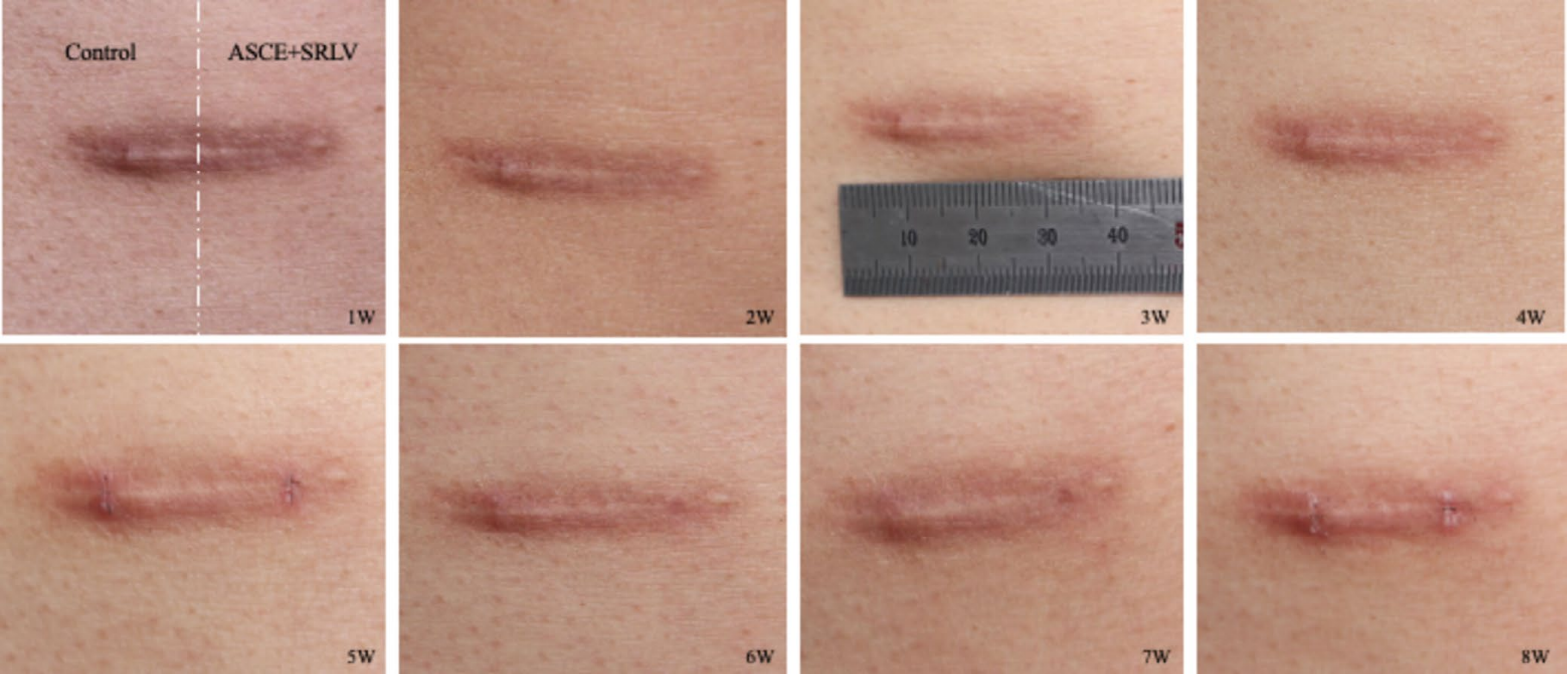
Changes in scars over time. The left side is the control group (HA), the right side is the experimental group (ASCE‐Exosome). The result photo shows from the left side to the right and from the bottom left to the right, the intervals of one week.

Representative clinical photographs of a postoperative linear chest scar are shown (Figure 2). Wound dehiscence requiring additional treatment was predefined as an exclusion criterion, but no such cases occurred, and no participants were lost to follow‐up during the study period.

Quantitative analysis was conducted up to 8 weeks, demonstrating significant improvements on the exosome‐treated side in pigmentation, pliability, and relief as evaluated by OSAS and VSS, while vascularity showed no significant difference (Video. 1).

**FIGURE 3:**
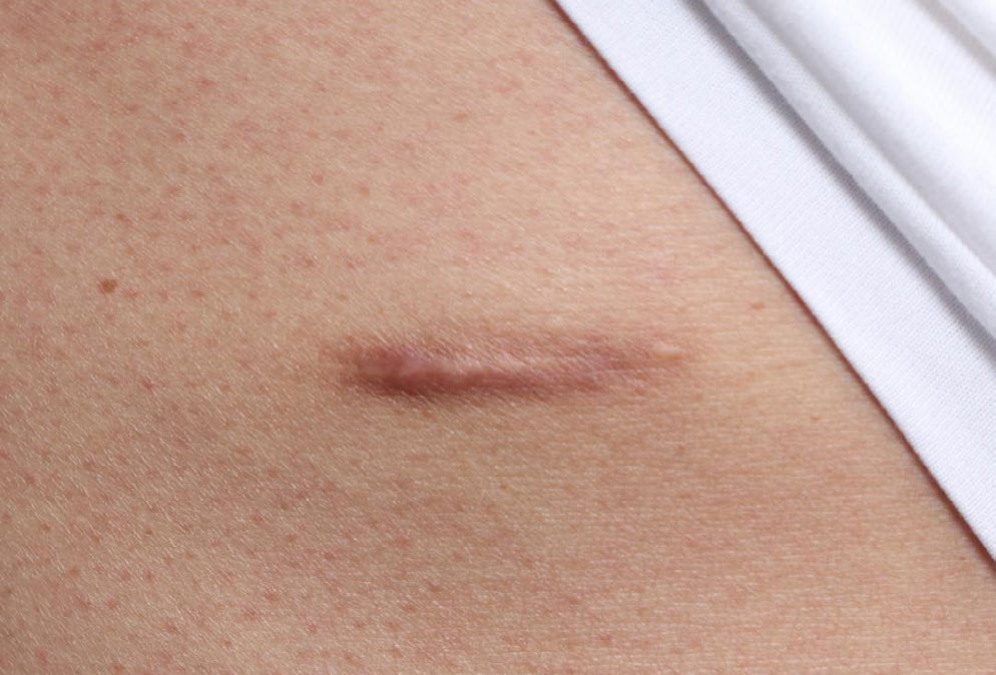
Longitudinal photographic assessment at 12 months demonstrated superior improvements in scar height and thickness on the exosome‐treated side.

To illustrate long‐term outcomes, additional clinical photographs were obtained in 1 year (Figure 3). These images confirmed that the improvements observed during the 8‐week evaluation—particularly in pigmentation, pliability, and relief—were consistent with sustained improvements in scar quality. Long‐term follow‐up also revealed significant reductions in scar height and thickness on the exosome‐treated side compared with the control side.

## 4 VSS And OSAS

### 4.1 Pigmentation

**FIGURE 4:**
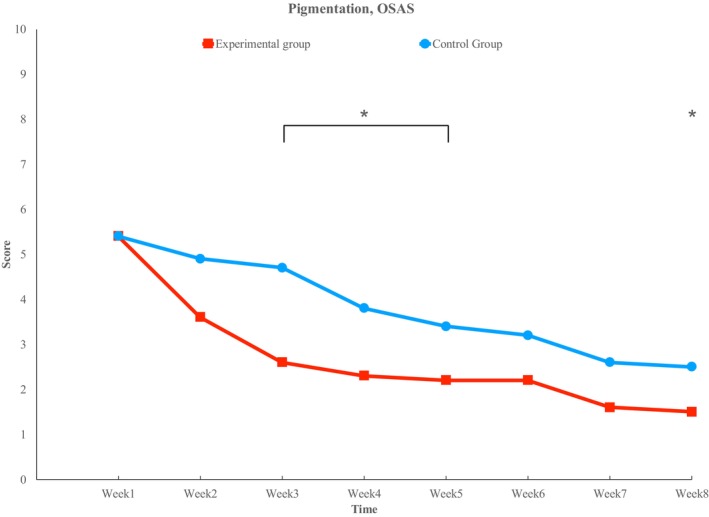
Pigmentation. Both sides shown improvement over time, but statistically significant improvement is observed in the experimental side at weeks 3,4,5 and 8. (*) indicates statistical significance (*p* < 0.05).

In the VSS group, no significant changes were observed in either the experimental or control sides. In the OSAS group, both the experimental and control sides showed a decreasing trend, with significant differences observed at week 3 (2.6 ± 1.51 vs. 4.7 ± 1.83), week 4 (2.3 ± 1.25 vs. 3.8 ± 1.99), week 5 (2.2 ± 1.14 vs. 3.4 ± 1.35), and week 8 (1.5 ± 0.97 vs. 2.5 ± 1.18) (*p* < 0.05) (Figure 4).

### 4.2 Pliability

**FIGURE 5:**
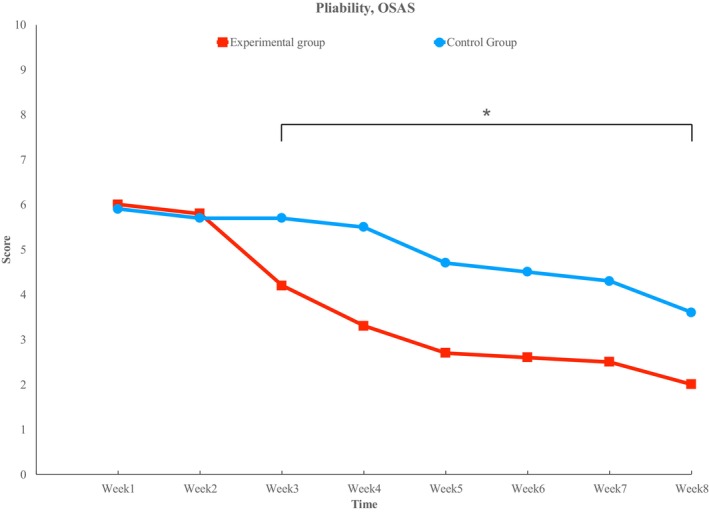
Pliability. There is a small decrease until week 2, but a difference begins to emerge from week 3, showing statistical significance from weeks 3 to 8. (*) indicates statistical significance (*p* < 0.05).

Both the VSS and OSAS groups showed a decreasing trend. In the VSS group, significant differences were observed at week 4 (1.4 ± 0.52 vs. 2.3 ± 0.67), week 5 (1.1 ± 0.32 vs. 1.8 ± 0.63), and week 6 (1.1 ± 0.32 vs. 1.8 ± 0.63). In the OSAS group, significant differences were observed at week 3 (4.2 ± 2.10 vs. 5.7 ± 1.95), week 4 (3.3 ± 1.77 vs. 5.5 ± 2.12), week 5 (2.7 ± 1.70 vs. 4.7 ± 1.95), week 6 (2.6 ± 1.43 vs. 4.5 ± 1.72), week 7 (2.5 ± 1.51 vs. 4.3 ± 1.57), and week 8 (2.0 ± 1.15 vs. 3.6 ± 1.58) (*p* < 0.05) (Figure 5).

### 4.3 Relief

**FIGURE 6:**
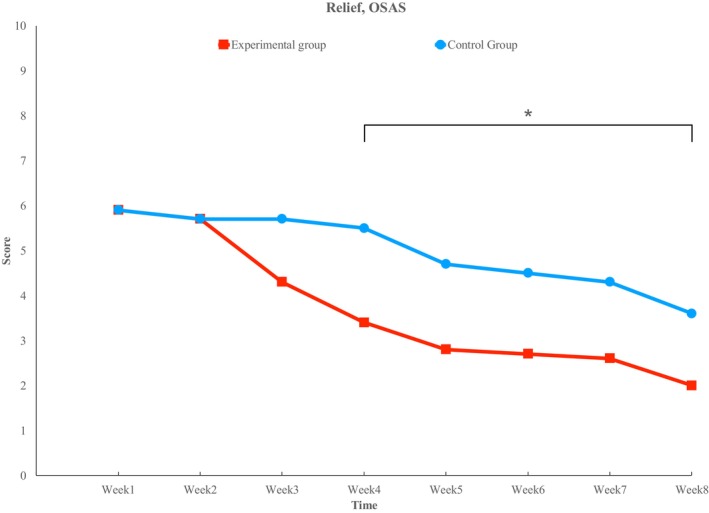
Relief. Starting from week 3, a trend difference between the two sides is observed, with statistical significance emerging from week 4. (*) indicates statistical significance (*p* < 0.05).

In the OSAS group, both the experimental and control sides showed a decreasing trend. Significant differences were observed at week 4 (3.4 ± 1.78 vs. 5.5 ± 2.12), week 5 (2.8 ± 1.69 vs. 4.7 ± 1.95), week 6 (2.7 ± 1.42 vs. 4.5 ± 1.72), week 7 (2.6 ± 1.51 vs. 4.3 ± 1.57), and week 8 (2.0 ± 1.15 vs. 3.6 ± 1.58) (*p* < 0.05) (Figure 6).

### 4.4 Height and Thickness

In the VSS group, no trend in height was observed over the 8‐week period, and no significant difference was observed between the experimental and control sides in terms of changes over time. Similarly, for thickness in the OSAS group, no trend was observed, and no significant difference was found (Tables 4 and 5).

**TABLE 4:** VSS score.

VSS								
Week	Pliability		Height		Vascularity		Pigmentation	
	Experimental	Control	Experimental	Control	Experimental	Control	Experimental	Control
1st	2.3 ± 0.67	2.3 ± 0.67	1.0 ± 0.67	1.0 ± 0.67	1.5 ± 0.85	1.5 ± 0.85	2.0 ± 1.05	2.7 ± 2.11
2nd	2.3 ± 0.67	2.3 ± 0.67	0.9 ± 0.74	1.0 ± 0.67	1.4 ± 0.70	1.4 ± 0.70	1.5 ± 0.71	2.5 ± 2.07
3rd	2.0 ± 0.47	2.3 ± 0.67	0.9 ± 0.74	1.0 ± 0.67	1.4 ± 0.70	1.4 ± 0.70	1.3 ± 0.48	2.4 ± 2.12
4th	1.4 ± 0.52*	2.3 ± 0.67	0.9 ± 0.74	1.0 ± 0.67	1.3 ± 0.67	1.3 ± 0.67	1.2 ± 0.42	2.2 ± 1.81
5th	1.1 ± 0.32*	1.8 ± 0.63	0.9 ± 0.74	0.9 ± 0.74	1.0 ± 0.94	1.1 ± 0.88	1.0 ± 0.47	1.9 ± 1.85
6th	1.1 ± 0.32*	1.8 ± 0.63	0.9 ± 0.74	0.9 ± 0.74	1.0 ± 0.94	1.1 ± 0.88	1.1 ± 0.32	2.1 ± 1.85
7th	1.1 ± 0.32	1.5 ± 0.53	0.9 ± 0.74	0.9 ± 0.74	1.0 ± 0.94	1.1 ± 0.88	1.2 ± 0.42	2.0 ± 1.89
8th	1.1 ± 0.32	1.4 ± 0.52	0.9 ± 0.74	0.9 ± 0.74	1.0 ± 0.94	1.1 ± 0.88	1.1 ± 0.32	1.9 ± 1.85

*Note:* An asterisk (*) denotes statistical significance (*p* < 0.05).

**TABLE 5:** OSAS score.

OSAS										
Week	Vascularity		Pigmentation		Thickness		Relief		Pliability	
	Experimental	Control	Experimental	Control	Experimental	Control	Experimental	Control	Experimental	Control
1st	3.8 ± 1.87	3.7 ± 1.77	5.4 ± 2.41	5.4 ± 2.41	6.0 ± 1.41	6.0 ± 1.41	5.9 ± 1.97	5.9 ± 1.97	6.0 ± 2.00	5.9 ± 1.97
2nd	3.6 ± 1.71	3.6 ± 1.71	3.6 ± 1.90	4.9 ± 2.08	5.3 ± 1.42	5.6 ± 1.35	5.7 ± 1.95	5.7 ± 1.95	5.8 ± 1.99	5.7 ± 1.95
3rd	3.5 ± 1.72	3.6 ± 1.71	2.6 ± 1.51*	4.7 ± 1.83	5.1 ± 1.52	5.5 ± 1.43	4.3 ± 2.06	5.7 ± 1.95	4.2 ± 2.10*	5.7 ± 1.95
4th	3.4 ± 1.71	3.5 ± 1.72	2.3 ± 1.25*	3.8 ± 1.99	4.8 ± 1.69	5.3 ± 1.42	3.4 ± 1.78*	5.5 ± 2.12	3.3 ± 1.77*	5.5 ± 2.12
5th	2.9 ± 1.66	3.2 ± 1.55	2.2 ± 1.14*	3.4 ± 1.35	4.6 ± 1.90	5.1 ± 1.45	2.8 ± 1.69*	4.7 ± 1.95	2.7 ± 1.70*	4.7 ± 1.95
6th	3.0 ± 1.56	3.2 ± 1.55	2.2 ± 1.32	3.2 ± 1.48	4.4 ± 2.07	5.1 ± 1.45	2.7 ± 1.42*	4.5 ± 1.72	2.6 ± 1.43*	4.5 ± 1.72
7th	3.0 ± 1.56	3.1 ± 1.52	1.6 ± 1.07	2.6 ± 1.35	4.3 ± 2.16	4.8 ± 1.69	2.6 ± 1.51*	4.3 ± 1.57	2.5 ± 1.51*	4.3 ± 1.57
8th	2.9 ± 1.66	3.1 ± 1.52	1.5 ± 0.97	2.5 ± 1.18	4.2 ± 2.25	4.7 ± 1.77	2.0 ± 1.15*	3.6 ± 1.58	2.0 ± 1.15*	3.6 ± 1.58

*Note:* An asterisk (*) denotes statistical significance (*p* < 0.05).

### 4.5 PSAS

No significant differences in pain and itching scores were observed between the experimental and control sides at any time point. Significant differences in color were found starting from week 4 (2.5 ± 1.58 vs. 4.1 ± 1.66). Significant differences in stiffness were observed from week 3 (3.3 ± 1.42 vs. 4.3 ± 1.25), while thickness scores showed significant differences from week 2 (3.4 ± 0.97 vs. 4.4 ± 0.97). Significant differences in irregularity were observed at week 4 (2.5 ± 1.08 vs. 4.0 ± 1.49) and 8 (2.4 ± 1.07 vs. 3.1 ± 1.29) (Table 6).

**TABLE 6:** PSAS score.

PSAS												
Week	Painful		Itching		Color		Stiff		Thickness		Irregular	
	Experimental	Control	Experimental	Control	Experimental	Control	Experimental	Control	Experimental	Control	Experimental	Control
1st	0.7 ± 0.48	0.7 ± 0.48	1.0 ± 1.25	1.0 ± 1.25	5.1 ± 1.60	6.0 ± 1.76	4.4 ± 1.51	4.4 ± 1.51	4.8 ± 1.14	4.7 ± 1.16	4.7 ± 1.25	4.6 ± 1.17
2nd	0.7 ± 0.48	0.7 ± 0.48	0.9 ± 0.99	1.0 ± 1.25	3.4 ± 1.43	5.0 ± 1.70	4.2 ± 1.23	4.4 ± 1.51	3.4 ± 0.97*	4.4 ± 0.97	4.2 ± 1.40	4.4 ± 1.26
3rd	0.7 ± 0.48	0.7 ± 0.48	0.8 ± 0.79	0.9 ± 0.99	2.8 ± 1.48	4.4 ± 1.90	3.3 ± 1.42*	4.3 ± 1.25	3.2 ± 1.03*	4.3 ± 0.82	3.3 ± 1.25	4.2 ± 1.40
4th	0.7 ± 0.48	0.7 ± 0.48	0.8 ± 0.79	0.9 ± 0.99	2.5 ± 1.58*	4.1 ± 1.66	3.0 ± 1.05	3.7 ± 1.49	3.1 ± 1.10	3.6 ± 1.07	2.5 ± 1.08*	4.0 ± 1.49
5th	0.2 ± 0.42	0.7 ± 0.48	0.3 ± 0.67	0.9 ± 0.99	2.3 ± 1.64*	3.6 ± 1.43	2.1 ± 1.10*	3.5 ± 1.35	2.4 ± 1.26*	3.4 ± 1.07	2.5 ± 0.97	3.4 ± 1.26
6th	0.2 ± 0.42	0.7 ± 0.48	0.2 ± 0.42	0.9 ± 0.99	2.3 ± 1.64	3.6 ± 1.58	1.9 ± 0.88*	3.4 ± 1.07	2.3 ± 1.34*	3.4 ± 1.07	2.5 ± 0.97	3.2 ± 1.32
7th	0.2 ± 0.42	0.2 ± 0.42	0.2 ± 0.42	0.3 ± 0.67	2.1 ± 1.66*	3.4 ± 1.65	1.8 ± 0.92*	2.8 ± 1.32	2.4 ± 1.35	2.9 ± 1.45	2.6 ± 0.97	3.3 ± 1.25
8th	0.2 ± 0.42	0.2 ± 0.42	0.2 ± 0.42	0.3 ± 0.67	1.8 ± 1.69*	3.2 ± 1.55	1.8 ± 0.92	2.7 ± 1.34	2.2 ± 1.40	2.6 ± 1.35	2.4 ± 1.07*	3.1 ± 1.29

*Note:* An asterisk (*) denotes statistical significance (*p* < 0.05).

## 5 Discussion

Scar management has long been a challenging task, with various methods explored over the years. Traditionally, steroid injections (triamcinolone) [1], silicone ointments, and sheets have been commonly used [2]. Additionally, energy‐based devices (e.g., CO2 and dye lasers) have been widely employed [4]. More recently, skin boosters such as polydeoxyribonucleotide and polynucleotide, or regenerative solutions such as exosomes, have emerged as treatment options [13].

Regenerative solutions, particularly exosomes, have been shown to aid in wound healing and anti‐inflammation, thereby diminishing scar formation [16]. While basic mechanisms are understood, many clinical effects, primarily related to acne or skin conditions, lack sufficient evidence in the context of surgical scars, making direct application and prediction challenging.

This study meticulously documented an 8‐week treatment period, revealing when and to what extent specific postoperative scar aspects improve. The most notable improvement was observed in pigmentation. Consistent with previous research, the lightening effect of the skin was evident [21], with a significant reduction in pigmentation of scars. Pigmentation‐related scars appear to be the most promising target for treatment.

Pigmentation is induced by melanocytes, and exosomes have been reported to directly and indirectly influence the metabolic pathways of these enzymes, thereby inhibiting melanin production [22]. More specifically, TGF‐β1 contained within exosomes reduces the expression of TYR, TRP‐1, and TRP‐2, leading to the suppression of melanin synthesis [21]. Exosomes modulate this signaling pathway, ultimately contributing to the improvement of hyperpigmentation. The formation of new melanin pigment typically takes approximately 48 to 72 h. If exosome‐based depigmentation treatment is administered at weekly intervals, this timing coincides with the turnover cycle of melanin synthesis, which occurs around 72 h. Therefore, theoretically, the primary observable effect following exosome‐based treatment is expected to be associated with pigmentation [23]. Although melanin production is expected to decrease after one week, resulting in reduced pigmentation, statistical significance in this study was observed from week 3. In the actual experiment, most patients on the experimental side reported noticing an improvement in pigmentation starting from week 2, but the statistical difference was observed only by week 4. There was a difference in scores between the two sides, and the score gap between them changed over time. Therefore, the effect appeared from the beginning, but statistically, a decreasing trend was observed over time, and by week 4, the score difference between the two sides became statistically significant. Similarly, other studies have reported no significant changes within the first two weeks, with noticeable improvements occurring between week 4 and week 8. Since melanin production must decrease while pre‐existing melanin pigments are also cleared, clinical efficacy tends to become apparent around week 3 [24].

Another significant improvement was observed in pliability. During the wound healing process, both collagen type I and type III play key roles at the surgical site. Collagen type I is responsible for maintaining tissue strength and stability, while collagen type III governs tissue elasticity [25]. In normal tissue, collagen type I makes up about 80%, and type III accounts for about 20%. Early in the wound healing process, collagen type III predominates, gradually being replaced by type I [26]. It is well known that a higher collagen type I/III ratio leads to more pronounced scar formation. This can be observed in the skin of infants, where a higher proportion of collagen type III is associated with reduced scarring [27].

In contrast to normal tissue, the wound healing process in scar tissue involves several stages where TGF‐β expression remains persistently elevated. This upregulation enhances fibroblast activity, increasing collagen production by approximately fourfold [28]. Exosomes regulate TGF‐β activity and modulate fibroblast function, contributing to improved scar remodeling. Consequently, collagen type I in the reticular dermis is regulated, contributing to better scar formation. This process occurs during the remodeling phase of wound healing maturation, which can take from 8 days to up to a year [26]. In this study, it is expected that exosomes would take approximately 2 to 3 weeks to regulate collagen production. By the third week, a noticeable improvement in pliability was observed, indicating the exosome's effect on collagen remodeling, particularly the regulation of α‐smooth muscle actin (α‐SMA), fibroblast growth factor‐2 (FGF‐2), elastin, and TGF‐β mRNA expression [29]. These findings were observed in animal experiments. Further molecular studies are required to clarify the specific effects of exosomes on COL1A1 and COL3A1 expression and their role in scar remodeling [30].

In this study, the focus was on surgical incision wounds rather than skin lesions, which resulted in minimal changes in parameters such as vascularity or thickness. These parameters are more effectively assessed in studies targeting skin lesions, and it is believed that using skin lesions as a model would yield more pronounced results in these aspects.

Despite these promising findings, several limitations must be acknowledged. First, hyaluronic acid (HA) was used as the control substance. HA has independent wound‐healing and scar‐modulating properties, which may have underestimated the comparative efficacy of exosomes. However, HA was chosen for its physical similarity to exosomes in appearance and viscosity, ensuring feasibility in a double‐blind study design. It should be emphasized that in actual clinical practice, the benefit from exosomes alone could be greater than observed in this study.

Second, all scars were treated with micro‐needle radio frequency (RF) prior to exosome or HA application. RF itself has a potential effect on scar remodeling, representing a confounding factor. This approach was selected because RF is commonly used in clinical practice to enhance absorption of topical agents and reflects treatment protocol. Nonetheless, the absence of micro‐needle RF‐only control group limits the ability to isolate the independent effects of exosomes. Future studies should incorporate additional control groups to address this.

Third, although this study employed an 8‐week quantitative follow‐up, long‐term outcomes are critical in scar management. To partially address this, standardized 1‐year follow‐up photographs were included, which demonstrated persistence of early improvements. Additionally, commonly used scar assessment methods such as VSS and POSAS are inherently subjective, reflecting their limited objectivity. Over time, it becomes difficult for patients to accurately recall their previous evaluations, making direct comparisons less reliable. Therefore, future studies should aim to obtain more objective results using techniques such as ultrasound or 3D skin analysis [31, 32]. To further clarify outcomes, the authors are planning histological examinations in subsequent studies. Although some previous studies have reported histological findings, they are limited to a single case, which is insufficient [33]. The authors are preparing a larger number of cases to obtain results that are at least clinically meaningful.

Finally, the relatively small sample size (*n* = 10) limits the generalizability of the findings. The small sample was due to the difficulty of recruiting surgical patients who were cooperative and able to attend weekly treatments. As a result, the timing of postoperative treatment varied widely, ranging from 1 to 11 months, which could introduce bias because the wound healing process progresses through different phases [34, 35]. Future studies with larger sample sizes would be valuable to investigate how exosomes affect wounds at different stages of healing. Despite these limitations, the split‐scar design strengthens within‐patient comparisons; however, this study should still be considered a pilot investigation. Larger, multicenter randomized controlled trials will be necessary to establish definitive clinical recommendations.

Taken together, these findings suggest that ASC‐exosomes improve pigmentation and pliability of postoperative scars, potentially through pathways involving melanin synthesis inhibition and collagen remodeling. Importantly, this study highlights the feasibility of incorporating exosomes into scar management protocols but also underscores the need for more rigorous methodological designs in future work.

## 6 Conclusion

This study demonstrated that ASC‐exosomes contribute to meaningful improvements in postoperative scar quality, particularly in pigmentation and pliability. The reduction in hyperpigmentation is likely due to exosome‐mediated inhibition of melanin synthesis, while improved pliability may reflect regulation of collagen remodeling, leading to softer and more natural tissue formation.

## Funding

The authors have nothing to report.

## Ethics Statement

This study was conducted in accordance with the principles of the Declaration of Helsinki.

## Consent

Written informed consent was obtained from all participants involved in the study.

## Conflicts of Interest

Dr. Dong‐Woo, Jung and Dr. Kui Yong, Park declare that this work received no financial support. The ASC‐exosome product used in this study was provided by ExoCoBio Inc. for research purposes. ExoCoBio Inc. also contributed to scientific discussions regarding the formulation but had no role in study design, data collection, data analysis, or manuscript preparation. The authors alone are responsible for the content and writing of this article.

Dr. Kyung Hwan, Jeong has received honoraria for speaking engagements for two conference lectures organized by the Dermatology Committee of Chinese Non‐Government Medical Institutions Association (CNMIA) and the Dermatology and Aesthetic Summit Forum of International Medicine of Anti‐aging & Aesthetics Congress (IMAAC), and two webinars organized by ExoCoBio Inc. for participating in educational purposes (online educational programs for physicians in China, Europe, the Middle East, Taiwan and Malaysia) These activities were independent of the present study and did not influence its study design, data collection, data analysis or conducting manuscript preparation.

## Supporting information


**Video S1:** This video demonstrates postoperative scar evaluation using the Vancouver Scar Scale (VSS) and Observer Scar Assessment Scale (OSAS). The assessment highlights vascularity, pliability and relief, emphasizing subtle changes in erythema, tissue elasticity, and surface contour that are not easily distinguished by visual inspection alone.

## Data Availability

The data that support the findings of this study are available on request from the corresponding author. The data are not publicly available due to privacy or ethical restrictions.
